# Surprising Use of the Business Innovation Bass Diffusion Model To Accurately Describe Adsorption Isotherm Types I, III, and V

**DOI:** 10.1021/acs.langmuir.3c00147

**Published:** 2023-03-13

**Authors:** Lukas W. Bingel, Krista S. Walton

**Affiliations:** School of Chemical and Biomolecular Engineering, Georgia Institute of Technology, Atlanta, Georgia 30332, United States

## Abstract

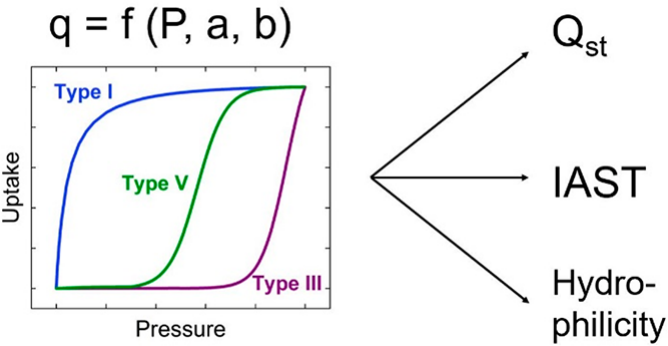

Using adsorption isotherm data to determine heats of adsorption or predict mixture adsorption using the ideal adsorbed solution theory (IAST) relies on accurate fits of the data with continuous, mathematical models. Here, we derive an empirical two-parameter model to fit isotherm data of IUPAC types I, III, and V in a descriptive way based on the Bass model for innovation diffusion. We report 31 isotherm fits to existing literature data covering all six types of isotherms, various adsorbents, such as carbons, zeolites, and metal–organic frameworks (MOFs), as well as different adsorbing gases (water, carbon dioxide, methane, and nitrogen). We find several cases, especially for flexible MOFs, where previously reported isotherm models reached their limits and either failed to fit the data or could not sufficiently be fitted due to stepped type V isotherms. Moreover, in two instances, models specifically developed for distinct systems are fitted with a higher *R*^2^ value compared to the models in the original reports. Using these fits, it is demonstrated how the new Bingel–Walton isotherm can be used to qualitatively assess the hydrophilic or hydrophobic behavior of porous materials from the relative magnitude of the two fitting parameters. The model can also be employed to find matching heats of adsorption values for systems with isotherm steps using one, continuous fit instead of partial, stepwise fits or interpolation. Additionally, using our single, continuous fit to model stepped isotherms in IAST mixture adsorption predictions leads to good agreement with the results from the osmotic framework adsorbed solution theory that was specifically developed for these systems using a stepwise, approximate fitting, which is yet far more complex. Our new isotherm equation accomplishes all of these tasks with only two fitted parameters, providing a simple, accurate method for modeling a variety of adsorption behavior.

## Introduction

The successful design and modeling of adsorption-based technologies for improving energy consumption of chemical separations require the knowledge of critical thermodynamic properties and mixture adsorption data.^1,2^ The existence of mathematical descriptions of adsorption isotherms using a single continuous model, i.e. a model that fits the whole isotherm, is critical for determining these design data. Quantification of adsorption affinities regarding regeneration and preferred adsorption through isosteric heats of adsorption using the Clausius–Clapeyron approach depends upon precise isotherm fits at several temperatures.^3^ Accurate predictions of mixture adsorption based on the most commonly used method to predict co-adsorption, the ideal adsorbed solution theory (IAST), rely on highly accurate pure-component adsorption fits, especially in the low-pressure region.^4^ In addition, assessing the reproducibility of repeated isotherm measurements is only possible with mathematical models describing the isotherms.^5−7^ For these applications, an accurate, simple, descriptive prediction of the adsorption data is more practical than a complex isotherm model with multiple fitting parameters even if the parameters provide a more quantitative description of system properties. Even more problematic, isotherm models developed for specific adsorbate/adsorbent systems are often limited in their extendibility to other systems with similar isotherm shapes due to a lack of knowledge of required properties for fitting parameters.

According to the nomenclature guidelines from both the first and updated IUPAC report,^8,9^ isotherms generally fall into six categories. Due to their unique shapes, the fitting capability of existing isotherm models is limited to only one or a few types. While traditional models such as Langmuir, Freundlich, and the Brunauer−Emmett−Teller (BET) model work well for types I, II, and IV,^10^ the recent surge in water adsorption research^11−13^ and the emergence of flexible materials^14^ with type V isotherms provide a myriad of examples where existing isotherm models have a limited ability to fit the curves. Thus, several models for specific systems with water vapor adsorbing in a type V shape have been developed (Table S2). They contain fitting parameters unique to the system describing physical properties ranging from site densities^15−17^ to Henry constants^17^ or functional group concentrations.^18^ Mahle and Friday developed a complex expression that contains the pore size distribution as a gamma function based on the Sircar isotherm.^19^ Mahle later modified this approach by incorporating an inverse tangent containing function.^20^ Stoeckli et al.^21^ demonstrated the applicability of the Dubinin–Astakhov model for S-shaped water adsorption in carbons, which is based on the energetic adsorption potential distribution.^22^ A cluster model for the stepped isotherm for carbon dioxide adsorption in the metal–organic framework IRMOF-1 was developed by Butyrskaya and Zapryagaev.^23^ Knowledge and applicability of all properties embedded in the models are required for their use, which limits their generalizability and sacrifices their general application as a fitting tool. This system restriction becomes even more critical with the development of novel materials with unique isotherm shapes where all traditional models reach their limits. One key example includes third-generation porous frameworks with structural flexibility. Their step-shaped isotherms^14^ indicate outstanding storage and capture properties^24^ as well as good separation ability^25^ but are difficult to fit for calculations with existing models. Yao et al. highlighted that binary co-adsorption predictions using IAST based on single-component isotherm models are not possible for these materials and suggested difficult mixture experiments^26^ as the only alternative.^27^ Interpolation between the isotherm points has been used in applications of IAST to get good fits in cases with stepped isotherms as it is even built into the commonly used pyIAST prediction tool.^28,29^ The lack of models for isotherms in these flexible adsorbents also limits the utility of heats of adsorption calculations based on the Clausius–Clapeyron equation. McGuirk et al. again used interpolation for their calculations in the flexible CdIF-13.^24^ Alternatively, separate fittings of the pre- and post-step region using traditional mathematical formulations have been used by Klein et al. for hydrogen in ZIF-7.^30^ This stepwise method also represents the key approach for the development of the osmotic framework adsorbed solution theory (OFAST) by Coudert.^31^ In other approaches, models have been derived as the second-order truncation of the general statistical thermodynamics isotherm by Ruthven^32^ or a modification of the Langmuir isotherm using a Fermi–Dirac distribution accounting for adsorbent–adsorbate interactions and surface heterogeneity.^33^ Ng et al. also extended the Langmuir isotherm to include fractional probabilities for the homotattic patch approximation and site energies.^34^ However, these generalizable models need complex solver mechanisms and include high numbers of fitting parameters.

The Bass model of innovation diffusion^35^ developed in the late 1960s describes the adoption and diffusion of an invention over time and has been widely used in market forecasting for both new product sales forecasting and technology forecasting. The simple two-parameter model combines the effects of early-adopters and word-of-mouth, resulting in curves that resemble either the type I adsorption isotherm shape or S-shaped curves. More details are given below in the Methods section.

In this paper, we derive a simple, empirical, descriptive isotherm model based on the Bass diffusion model and show its versatility for adsorption applications in terms of fitting isotherms of various adsorption systems and isotherm shapes as well as assessing material characteristics and determining critical adsorption properties based on these isotherm data.

## Methods

### Derivation of the Isotherm Model

The derivation of the presented isotherm model qualitatively follows the approach used to develop the initial Bass diffusion model.^35^ Here, we translate it to the adsorption of gases in porous materials. The original nomenclature is adjusted to represent known quantities in the adsorption field and prevent misinterpretation.

In the derivation by Bass, the number of adopters *q* in the time period between *p* and *p* – 1 results from two main contributions as shown in eq 1:
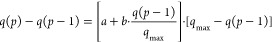
1

The parameter *a* reflects the impact of innovators independent of the previous buyers, whereas *b* describes the influence of imitators and purchases due to word-of-mouth. The expression in the last set of brackets represents the number of possible customers that have not adopted yet and can be attracted before saturation of the market capacity *q*_max_. A qualitative assessment of the relative magnitudes of the two parameters indicates the innovation diffusion type. Large *a* values correspond to a low-risk innovation with intrinsically fast adoption resulting in inverse J-shaped curves, while a larger *b* value represents S-shaped curves stemming from very new innovations^36^ that rely on only a few initial adopters until the product suddenly spreads due to community effects. Applied to adsorption, early-adopters can be seen as the intrinsic high-adsorption affinity of the surface engendered by specific adsorption sites. The word-of-mouth contribution of slower innovations refers to adsorption mechanisms that are mainly driven by molecules of the same species that are already adsorbed and thus present in the adsorbed phase. Here, the expression *q*_max_ – *q*(*p* – 1) represents the pore space not occupied until the saturation capacity *q*_max_ is reached. When using this model to fit isotherm data and saturation is not reached within the experimental range, the highest loading measured must be used; it is not a fitted parameter and is represented in units of an adsorbed quantity per weight of an adsorbent.

Starting from eq 1, the independent variable in translated from a time dependence into a pressure space and the non-linear differential equation d*q*/d*p* is solved leading to eq 2 using an assumption that *q* (*p* = 0) = 0 of no coverage at zero pressure to find the integration constant. The resulting Bingel–Walton equation is an extensive description of the loading *q* for a given pressure *p* of the isotherm. The mathematical step-by-step derivation is shown in the original publication by Bass.^35^
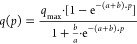
2

If detailed knowledge about the properties of the adsorbent and the adsorption site availability and distribution is available, this model can be extended or implemented in a more generic multi-site adsorption model. Here, the different site contributions can either be modeled using a sum of individual expressions based on eq 2 or other traditional isotherm models for each available adsorption site type.

For application in this work, the single site expression shown in eq 2 has been used. This isotherm equation requires that *a* + *b* > 0 to guarantee the continuously increasing shape of isotherms. When the pressure approaches zero, the model gives a Henry coefficient of *a*·*q*_max_. When the pressure goes to infinity, the limit is defined as the highest measured loading *q*_max_.

Following the Bass diffusion model, three assumptions are made.

1. Both adsorption mechanisms can happen at all pressure points. The key distinction is how they influence each other. Adsorption to specific sites is independent of the number of previously adsorbed molecules and only determined by the strength and availability of the adsorption sites in case of heterogeneously distributed sites. Mechanisms like pore filling, capillary condensation, and cluster formation depend on the number of already adsorbed molecules.
2. The importance of the first adsorbed molecules will be greater at first and diminish monotonically over time.
3. The parameter *a* is the intrinsic adsorption affinity between the adsorbate and the adsorbent (type I isotherm); *b* is the clustering coefficient describing strong adsorbate–adsorbate interactions (type III and V isotherms). Both parameters are in units of inverse pressure.

Since type V isotherms are not only common in the systems with strong intermolecular interactions of the same species but also observed in flexible MOFs with gate-opening behavior, here the coefficient *b* can be seen as a factor representing the local stress that is induced by adsorbed molecules driving the phase transition^37,38^ allowing for adsorption.

### Application to Literature Examples

To test the newly derived model, we applied it to a variety of cases reported in the open literature. The 31 sets of adsorption data cover all six types of isotherms according to the IUPAC nomenclature, different units for the pressure and uptake axes, and systems in terms of materials (carbons, zeolites, and MOFs) and adsorbing gases (carbon dioxide, nitrogen, water vapor, and methane). Raw isotherm data that were not tabulated in the original publication were digitized manually using digitization software. This introduces a systematic error, especially in figures with low resolution or large symbols, where it is difficult to find the exact center. The optimization of the fitting parameter was conducted using a minimization of the sum of squared errors in Python.

## Results and Discussion

**Figure 1:**
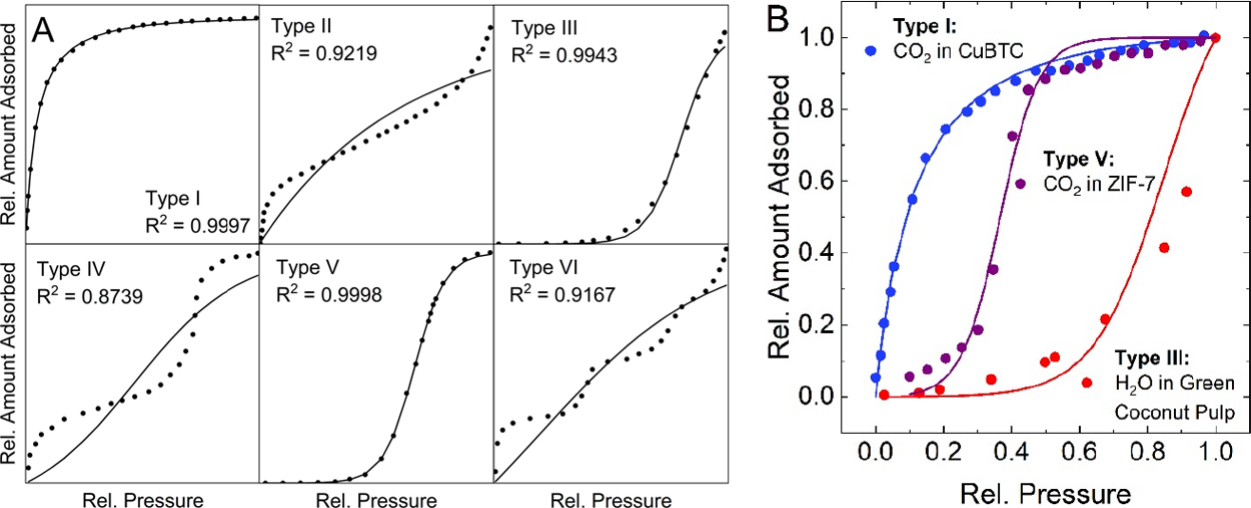
Fits as solid lines compared to data points for different isotherm types. (A) Fits to randomly sampled points of the examples to define the six different isotherm types in the IUPAC report.^9^ Quality of fits is assessed by the shown *R*^2^ values, and fitting parameters are listed in Table S3. (B) Fits of experimental data from the literature in relative units on both axes using the presented model for carbon dioxide in CuBTC at 298 K,^39^ carbon dioxide in ZIF-7 at 291 K,^40^ and water vapor in green coconut pulp at 343 K.^41^ Fitting parameter and coefficients of determination for the not-normalized fits are tabulated in Table S4. The corresponding plots in absolute units are given in Figure S2.

In its original application for innovation diffusion, the derived model describes inverse J-shaped and S-shaped curves. To test the general applicability of the model to the isotherm shapes defined in the updated 2015 IUPAC report for reporting isotherm measurements, randomly sampled points on the original example curves from the definitions were fitted using the newly derived expression. As shown in Figure 1A, these fittings result in accurate descriptions of type I, III, and V isotherms based on the shown coefficients of determination highlighting the general shapes the model is capable of modeling. For isotherms of types II, IV, and VI a trend of fitting one convex curve not representing the isotherm shape can be observed.

Since the points used in these examples are idealized from definition isotherms, we also applied the model to experimental data from the literature representing the three cases with successful fits as shown in Figure 1B. The curves are plotted in relative units normalized by the maximum value of each axis to make the trends more visual due to the different pressure and loading ranges covered by the examples. The original curves without normalization and additional experimental cases, including all isotherm types, are shown in Figures S1 and S2. Generally, the resulting *R*^2^ values are slightly lower due to fluctuations in experimental data compared to theoretical, simulated model data. The fit for type I adsorption of carbon dioxide in CuBTC results in an *R*^2^ of 0.998 confirming the good fitting capability. For the type V isotherm example of carbon dioxide adsorption in ZIF-7, a decrease down to 0.990 is observed. The model presented here can capture the position and steepness of the isotherm step well. However, it shows slight inaccuracies for the points right before and after the step if the step is not very sharp. The same observation can be made for other systems with type V isotherms as shown in Figure S1. A system with a very well defined, sharp step and very high resulting accuracy will be discussed later. Similar results can be found for the experimental type III isotherm of water vapor in a hydrophobic carbon material. The general type III shape can be represented. However, some accuracy within the step is lacking. Low accuracy again is found for the three other isotherm types when using experimental data (Figure S1). In particular, the trend described above of a convex model not representative of the isotherm shapes can be observed. Carbon dioxide in MIL-53(Fe) (Figure S1) shows a relatively high coefficient of determination at 89%. However, the two steps of the isotherm are not represented at all. Overall, the good results for types I, III, and V highlight the versatility and applicability of the derived model for a fast fitting of larger isotherm example sets for screening applications without model selection when only a descriptive fit with high accuracy is required. Such a fitting is especially advantageous for data sets containing stepped isotherms.^28^

**Figure 2:**
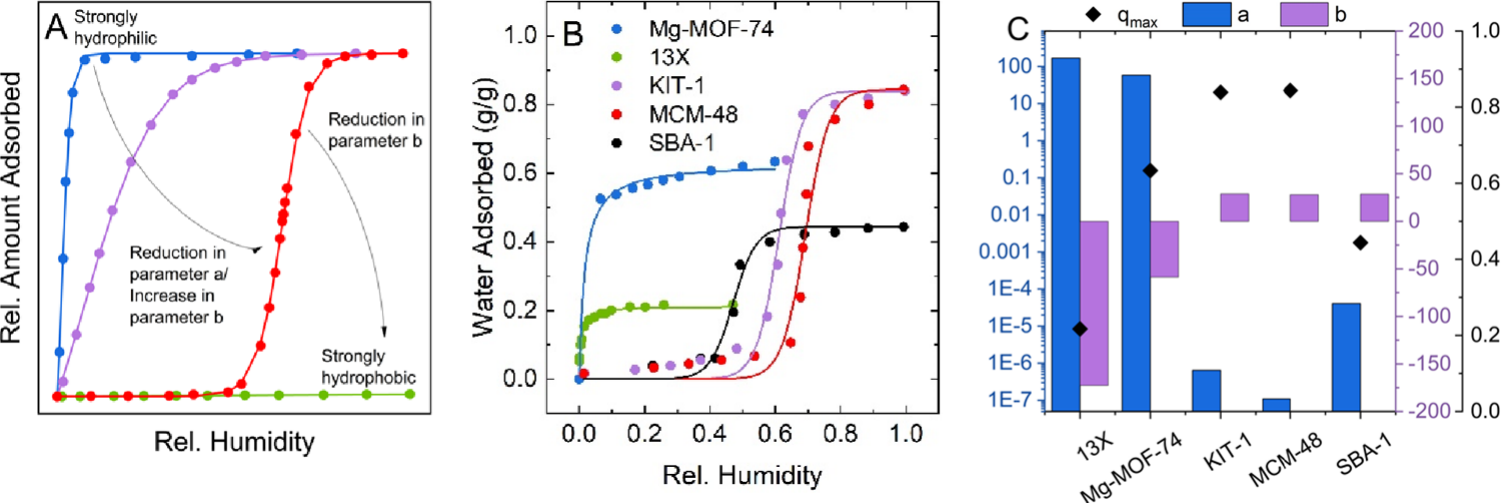
Application of the presented model to isotherms with different degrees of hydrophilic/hydrophobic properties. (A) General trend of the isotherm shape transitioning from strongly hydrophilic (type I) to hydrophobic (type V or almost no uptake). (B) Fits of the isotherm model to experimental literature data from five materials with different hydrophilicities.^42−44^ (C) Fitting parameters for the fits of the five experimental isotherms. The parameters *a* and *b* are unitless. The expression *q*_max_ is in units of g/g. Numerical values are tabulated in Table S5.

Water adsorption has attracted significant research attention due to its importance in applications, such as co-adsorption during carbon dioxide capture,^11^ atmospheric water harvesting,^12^ or structural degradation in novel materials impacting their stability.^13^ Key properties here are the hydrophobicity and hydrophilicity of the materials that can be qualitatively assessed from the shape of the isotherm.^13,42^ As shown in Figure 2A, they change from type I isotherms for (strongly) hydrophilic materials to type V isotherms for more hydrophobic materials until no uptake can be observed anymore in very hydrophobic, non-wetting structures. These general isotherm types can be represented by the model derived in this work. Using experimental data from the open literature that represent these different degrees of water affinity, fits were generated and are plotted in Figure 2B. The isotherm for the strongly hydrophilic zeolite 13X^43^ shows an inverse J-shape. As seen from the bar plot in Figure 2C, the isotherm is mainly dominated by the contribution from parameter *a*, the strong intrinsic water adsorption affinity, while the clustering parameter *b* has a negative contribution, meaning that it does not determine the isotherm shape. The fit of the slightly less hydrophilic Mg-MOF-74^44^ shows a reduction in the magnitude of *a*, while *b* becomes less negative approaching zero. With the three zeolites KIT-1, MCM-48, and SBA-1,^42^ a shift from isotherm type I to V occurs. It is reflected in a drastic reduction over orders of magnitude in the parameter *a*, while *b* becomes dominant. Interestingly, *b* remains almost unchanged for the three, generally describing the presence of a sharp step while *a* changes. In particular, comparing KIT-1 and MCM-41 with comparable *q*_max_ and *b* values and almost identical curves shifted along the *x* axis, *a* is reduced significantly. Once the step is present, *a* impacts the position of the step. This trend is confirmed when comparing the two materials just discussed to SBA-1, which has a similar sharpness but lower saturation capacity and shows a shift to the left along the *x* axis, which is again accompanied by a change in the *a* parameter that increases in this case.

In general, type I isotherms are dominated by parameter *a* with negative contributions from *b*. In contrast, type V isotherms have a positive contribution from coefficient *b* to reflect the presence of the isotherm step. Here, coefficient *a* determines the position of the step, which increases as it moves to higher pressures. This behavior provides the possibility of deriving simple screening descriptors for machine learning applications focused on the development of water adsorbing and/or repelling materials.^45^

**Figure 3:**
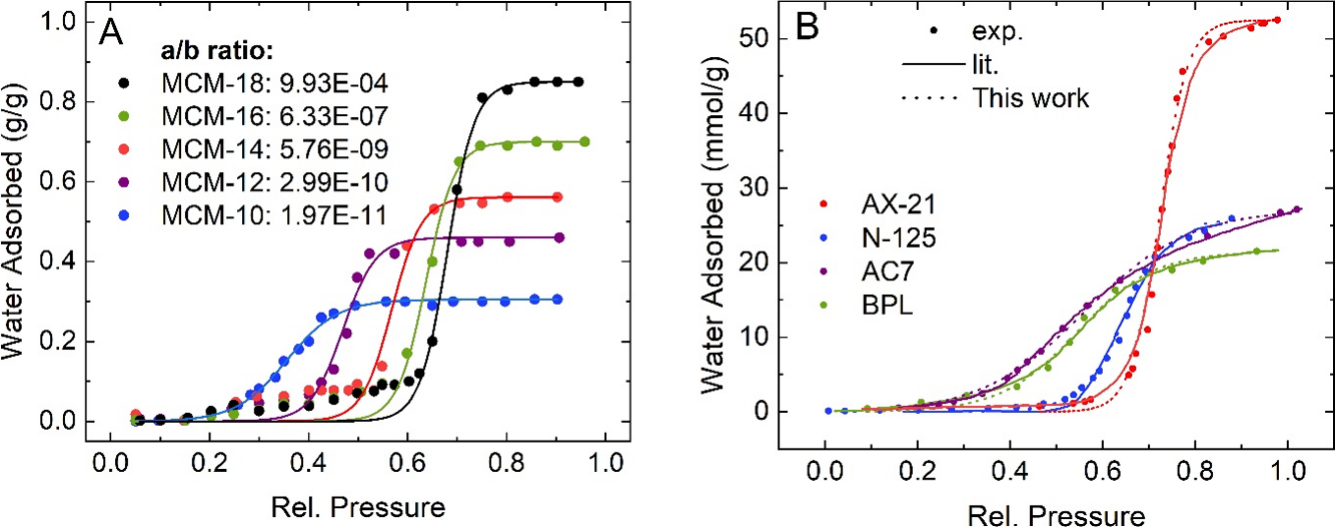
Fits to water isotherms from the literature. (A) Water isotherm fits for templated MCM-41 derivatives with different pore sizes synthesized and measured by Jähnert et al.^46^ The unitless ratio of the fitting parameters *a*/*b* decreases as the pore size is reduced. (B) Comparison of models derived for water adsorption in different carbon materials and the isotherm expression presented in this work. Experimental data are shown as symbols, literature fits as solid lines, and fits from the model from this work as dotted lines. Materials include the activated carbon AX-21 at 298 K,^17^ the carbon N-125 at 293 K,^21^ the microporous activated carbon AC7,^15^ and the activated carbon BPL carbon at 298 K.^20^ Fitting parameter and *R*^2^ values for both subplots are listed in Tables S6 and S7.

Jähnert et al. synthesized templated derivatives of the silica MCM-41^46^ with different, uniform pore sizes. The resulting water isotherms plotted in Figure 3A show steps of different magnitudes shifted along the pressure axis. They were fitted with the presented model with *R*^2^ values of at least 99%. The values are tabulated in Table S6 together with the fitting parameters. As the isotherms increase in saturation loading, the adsorption step occurs gradually with an onset at higher relative pressures. In these cases, the fit accuracy decreases slightly in this pre-step region, but the step shape and position as well as post-step points are fitted with high accuracy. Since the silica material is the same for all derivatives, it can be assumed that their hydrophilicity is constant and the shift in the isotherm step is solely due to the increased pore size. Utilizing the fitting parameters, we overall observed a drastic reduction of the ratio of *a* over *b* with increasing pore size. While *b* almost remains on a constantly high level representing the presence of the isotherm step, the parameter *a* decreases by orders of magnitudes for each step of the increased pore size. Here, the magnitude of parameter *b* indicates a similar pore condensation effect for all materials mainly controlled by intermolecular interactions between water molecules. However, the condensation depends on the nucleating monomer formation of adsorbed water molecules whose capillary effect decreases with the increased pore size reflected in the drastic decrease of parameter *a*. Here, the ratio of the two parameters presents an inverse correlation to the pore size if the hydrophilicity remains unchanged.

Most models developed for specific systems focus on water vapor as an adsorbate as it is known for its cluster formation and pore condensation, resulting in stepped isotherms. Figure 3B shows a comparison of four model examples from Table S2 of water adsorption in carbons compared to the model introduced here. Using this new expression, good agreement is observed for all four cases with coefficients of determination exceeding 0.99 in all cases. For the activated carbon AX-21, the *R*^2^ of 0.993 from the model presented in this work even exceeds the literature value of 0.991.^17^ The same is the case for the carbon N-125 with 0.996 in this work compared to 0.995 using the original Dubinin–Astakhov model.^21^ All three models have the same number of fitting parameters. In applications, such as calculations of heats of adsorption or reproducibility assessment, this higher accuracy combined with fast modeling without model selection is beneficial.

A second class of adsorbents and systems with increased research attention is flexible MOFs.^14^ Due to their gate-opening mechanism, they show stepped isotherms that are suitable candidates for gas capture and storage applications based on the increased usable capacity for adsorption and desorption cycles over a narrow pressure range.^24^ Moreover, the high dependence of the gate-opening step position on the adsorbing species indicates outstanding selectivity values when applied under mixture conditions.^25^ Quantification and investigation of both topics again require continuous fits for heats of adsorption calculations using the Clausius–Clapeyron equation or applying IAST for mixture predictions. However, the lack of a general isotherm model that accurately describes the step hinders successful predictions.^27^

**Figure 4:**
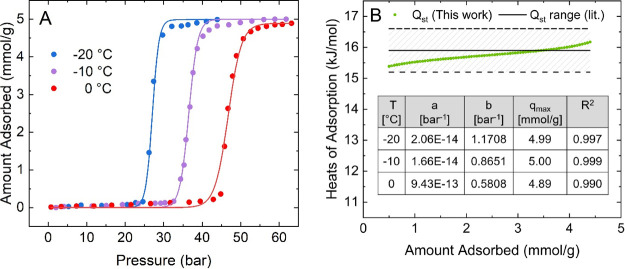
Clausius–Clapeyron approach to determine isosteric heats of adsorption to quantify the adsorption affinity of methane in CdIF-13. (A) Isotherm fits as continuous lines for experimental data (solid circles) from McGuirk et al.^24^ using eq 2 at −20, −10, and 0 °C. (B) Calculated heats of adsorption in the step loading range (green symbols) compared to the range reported in the original reference of Δ*H*_ads_ = −15.9 ± 0.7 kJ/mol. The inset shows the fitting parameters and quality of fits as tabulated in Table S8.

Figure 4A shows the example of methane adsorption in the flexible MOF CdIF-13 at three evenly spaced temperatures. The experimental points were fitted using the model presented in eq 2. The fitting parameters and *R*^2^ values are shown in the inset of Figure 4B. The latter present the high accuracy of the fits meeting and exceeding the commonly required 0.99.^5^ The model works well here due to the very sharp, distinct step in the isotherm without the gradual onset in the pre-step region as discussed above for Figure 3.

For the implementation and application of adsorbents, a key consideration is the adsorption affinity assessable through the isosteric heats of adsorption. The most commonly and easily used quantification approach based on the Clausius–Clapeyron equation relies on mathematical fits of the measured adsorption space to determine isobars. In the original publication of the system considered here, McGuirk et al. used interpolation to determine the heats of adsorption.^24^ As discussed above, compared to simple interpolation, the usage of *a* and *b* provides some quantitative information about the isotherm step position and sharpness. Then, employing the Clausius–Clapeyron approach using the three fits over a step loading range of 0.5–4.5 mmol/g, the heats of adsorption are determined and plotted in green in Figure 4B against the defined range from the original publication. The calculated values agree well with the range of Δ*H*_ads_ = −15.9 ± 0.7 kJ/mol.

Alternative approaches to determine heats of adsorption reported in the open literature utilize two continuous models. The inflection point is used to split the data set into two parts. Data points before and after the step are fitted separately, skipping points within the step.^30^ Using the model presented in this work shows an improvement over this approach as well since all points are considered and fitted accurately by a single equation. The same approach can be applied to determine adsorption strengths from stepped water isotherms as discussed above. Quantification of the binding affinity of water has important implications for choosing adsorbents for separations such as carbon dioxide removal. Water also strongly binds to high-energy adsorption sites requiring increased heats of regeneration during desorption that can be assessed using this approach.^47^

Looking at the isotherms shown in Figure 4A, it is also observable that with increasing temperature, the isotherm step is shifted to the right while keeping the shape, steepness, and saturation capacity constant. This trend is accompanied by parameter *a* being almost constant and approaching zero since there are no strong, intrinsic adsorption sites available at low pressures in the collapsed structure. At the same time, the parameter *b* decreases as the step shifts to higher pressures. Here, the latter can be seen as a qualitative, relative descriptor to define the stress that is needed to drive the gate-opening pressure for the same system at different temperatures, assuming that the shape and saturation capacity remain constant within the considered temperature range.

**Figure 5:**
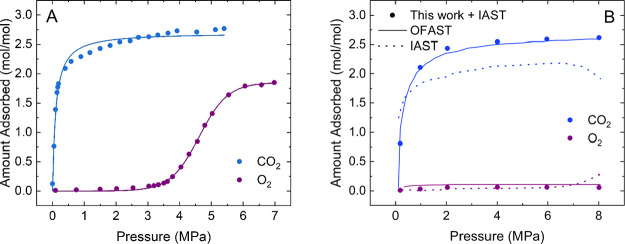
IAST predictions for the binary system of carbon dioxide and oxygen in Cu(dhbc)_2_(4,4′bpy) at 298 K. (A) Fits to single-component isotherms for experimental data from Kitaura et al.^49^ using the model derived in this manuscript. The fitting parameters and *R*^2^ are given in Table S9. (B) Comparison of mixture predictions using the model derived in this work combined with IAST, OFAST predictions, and direct IAST predictions using common isotherm models. The latter two predictions are from the work of Fraux et al.^48^

Yao and coworkers explained why regular IAST is not applicable to mixtures with components with stepped isotherms.^27^ Coudert developed the OFAS theory based on the approach of utilizing two independent models for fitting the isotherm before and after the step.^31^ An application of this method can be found in the literature for the co-adsorption of carbon dioxide and oxygen in Cu(dhbc)_2_(4′4-bpy), where the latter adsorbate shows a stepped isotherm.^48^ The experimental isotherms from Kitaura et al.^49^ were fitted using eq 2. The resulting mathematical descriptions are shown as solid lines compared to the experimental symbols in Figure 5A. They were implemented to solve the IAST equations to determine pressure-dependent loadings for the equimolar binary system over a pressure range of 0–8 MPa at 298 K, as shown in Figure 5B. The predictions of the individual loadings at six pressure points are shown as symbols. The solid lines of the OFAST calculations show a more realistic improvement over the regular IAST predictions (dotted lines) employing traditional isotherm models from the original work by Fraux and coworkers.

The results from this work show excellent agreement with the OFAST results shown as solid lines. The predictions for oxygen are slightly lower overall but follow the same trend and shape over the whole pressure range as compared to the unrealistic increase for O_2_ using IAST. The predictions also do not show the unreasonable sudden drop in the carbon dioxide uptake predicted using IAST around 7 MPa. The explanation for this decrease is the unrealistic assumption in IAST that oxygen does not have access to the open structure after carbon dioxide drives the phase transition until it reaches its transitioning pressure, which is not the case in the approach developed in this manuscript. Moreover, it results in realistic selectivity values of the order of 10s as compared to 100s using IAST around the gate-opening range.

Compared to OFAST, using the equation derived here means that a single model can be used to fit the whole isotherm with two parameters that contain relative information about the isotherm step and shape. Moreover, this one model can be easily implemented in commonly used tools, such as IAST++^50^ and pyIAST,^29^ avoiding interpolation and the computationally heavy numerical quadrature to determine the spreading pressures.

## Conclusions

In this paper, we have developed a new, descriptive isotherm model. The sigmoid-type expression describes three different types of IUPAC isotherms (I, III, and V) with high accuracy of *R*^2^ > 99% for a variety of experimental examples using only two fitting parameters *a* and *b* and the experimentally determined saturation capacity *q*_max_. Limitations and inaccuracies of the model occur in transition cases that are mixtures of different isotherm types and show a shallow step with an incremental increase in the pre-step region for type III and V isotherms. In particular, the new Bingel–Walton isotherm model allows for a continuous, mathematical description of general type V isotherms, which appear in novel flexible MOFs or many water adsorption cases. Compared to existing general models with fitting capability for type V isotherms, the equation presented here provides an easier fit due to the low number of parameters and an easier optimization of the same. Utilization of the fit expressions was shown for several key applications of adsorption technologies, such as calculations of heats of adsorption or IAST predictions for mixture adsorption. They can also be used for comparison of repeated measurements to assess their reproducibility. For water adsorption, the fitting parameters presented in this model also reveal relative information about the hydrophilicity of the adsorbent providing potential for machine learning-based screening of hydrophilic or wettable materials. Overall, the two parameters present a qualitative meaning about the type of interactions present in the system. A relatively high ratio of *a*/*b*, where *a* responds more drastically to changes of the isotherm, indicates that strong adsorbent–adsorbate interactions are present from sites with intrinsically high affinity. On the other hand, a relatively low quotient of *a*/*b* and *b* dominating the isotherm shape reflects the systems with strong adsorbate–adsorbate interactions stemming from cluster formation or adsorption-induced stress for a gate-opening phase transition. However, the magnitude of the ratio must be treated with care due to the high dependence of the units used for the adsorption pressure and uptake. An accurate fitting capability for flexible MOFs with sharp isotherm steps was presented, resulting in heats of adsorption identical to the results from interpolation. More systematic, well-controlled studies on specific systems are needed to explore the possibility of extracting quantitative, physical information about the system from the fitting parameters directly. Nevertheless, this new isotherm model offers clear advantages over other models with its capability of describing type I, III, and V isotherms, simplicity of two fitted parameters, general ease of use, and ability to capture model adsorption in flexible materials.

## Supporting Information Available

Summary of isotherm models; additional fits to literature examples; tabulated fitting parameters and coefficients of determination for isotherms fitted in this work (PDF)
