# Supplementary material for: Surprising Use of the Business Innovation Bass Diffusion Model To Accurately Describe Adsorption Isotherm Types I, III, and V
Source: Langmuir. 2023 Mar 13;39(12):4475–82. doi: 10.1021/acs.langmuir.3c00147 (PMC10061921; doi:10.1021/acs.langmuir.3c00147)
Supplement: Supplementary file 1 — la3c00147_si_001.pdf [file la3c00147_si_001.pdf]

Supporting Information for

Surprising Use of the Business Innovation Bass Diffusion Model to Accurately Describe Adsorption Isotherm Types I, III, and V

Lukas W. Bingel and Krista S. Walton[*]

School of Chemical & Biomolecular Engineering

Georgia Institute of Technology, Atlanta, GA 30332, United States

Corresponding Author

[*] E-Mail: krista.walton@chbe.gatech.edu.

KEYWORDS: adsorption, metal-organic framework, isotherm

Table of Contents

Figures

Figure S1 S3

Figure S2 S5

Tables

Table S1 S6

Table S2 S6

Table S3 S8

Table S4 S8

Table S5 S8

Table S6 S8

Table S7 S9

Table S8 S9

Table S9 S9

References S10

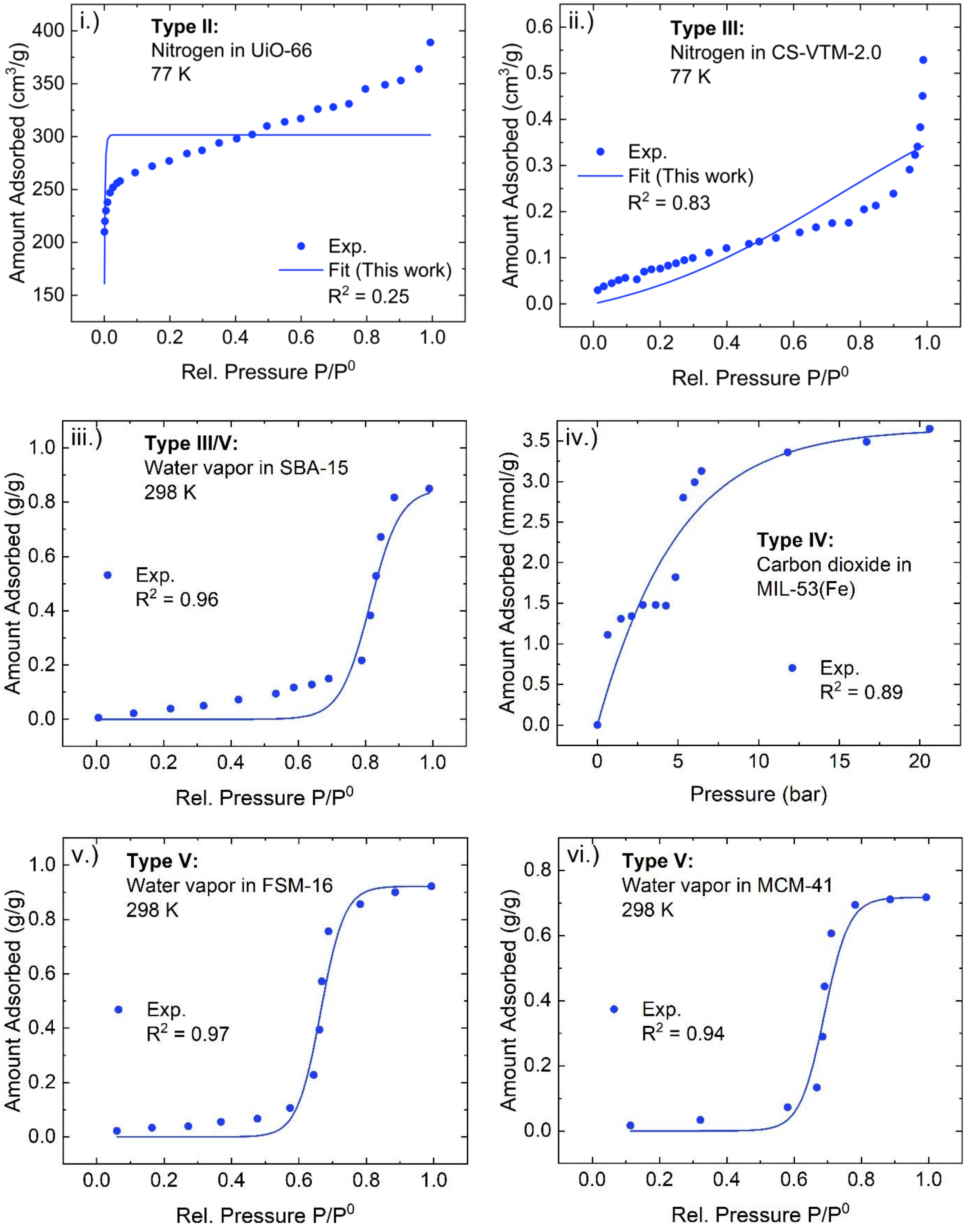

Figure S1. Examples from literature with isotherm fits using the model presented in this work. Fitting parameters, coefficients of determination and references are given in Table S1.

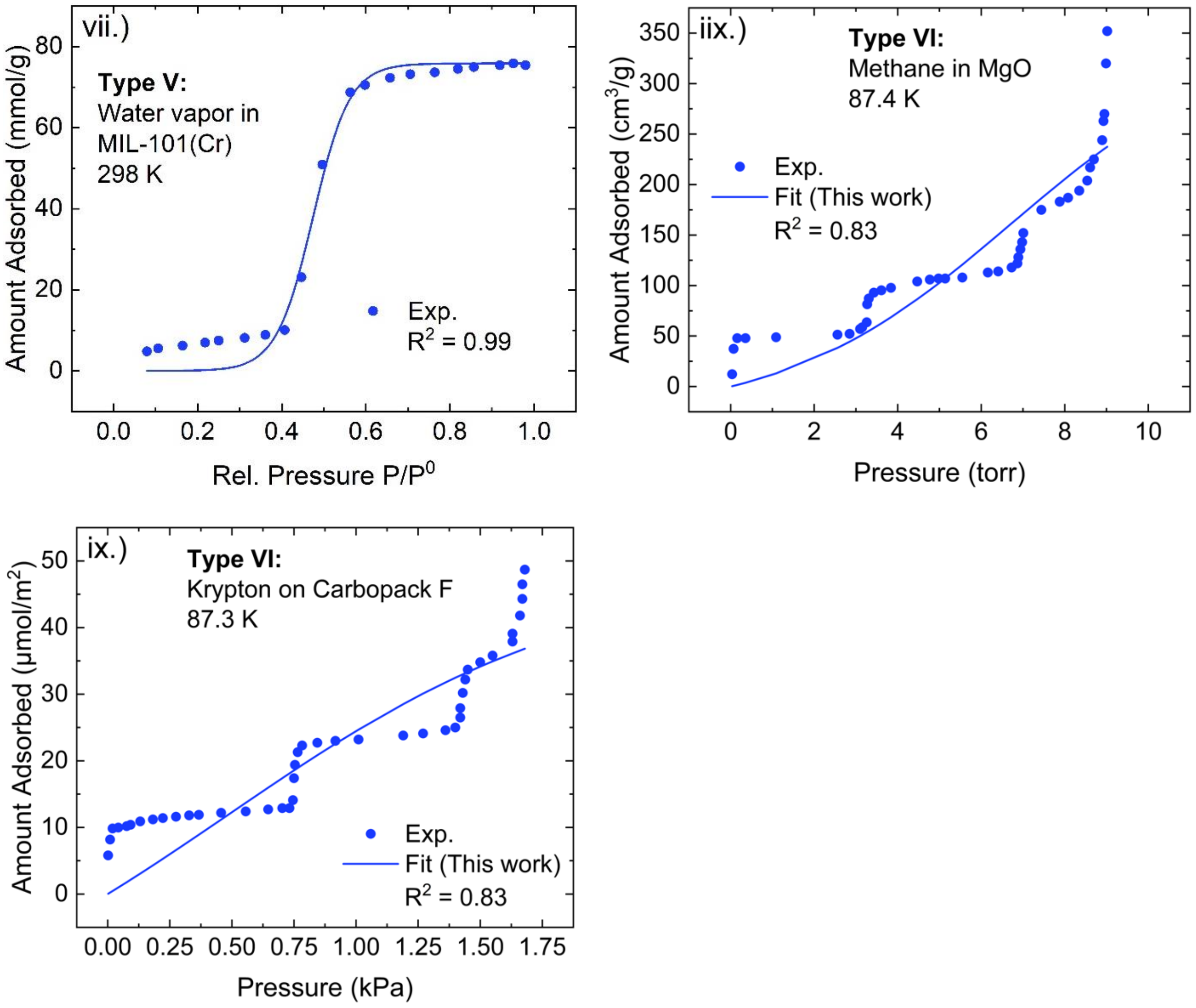

Continued **Figure S1.** Examples from literature with isotherm fits using the model presented in this work. Fitting parameters, coefficients of determination and references are given in Table S1.

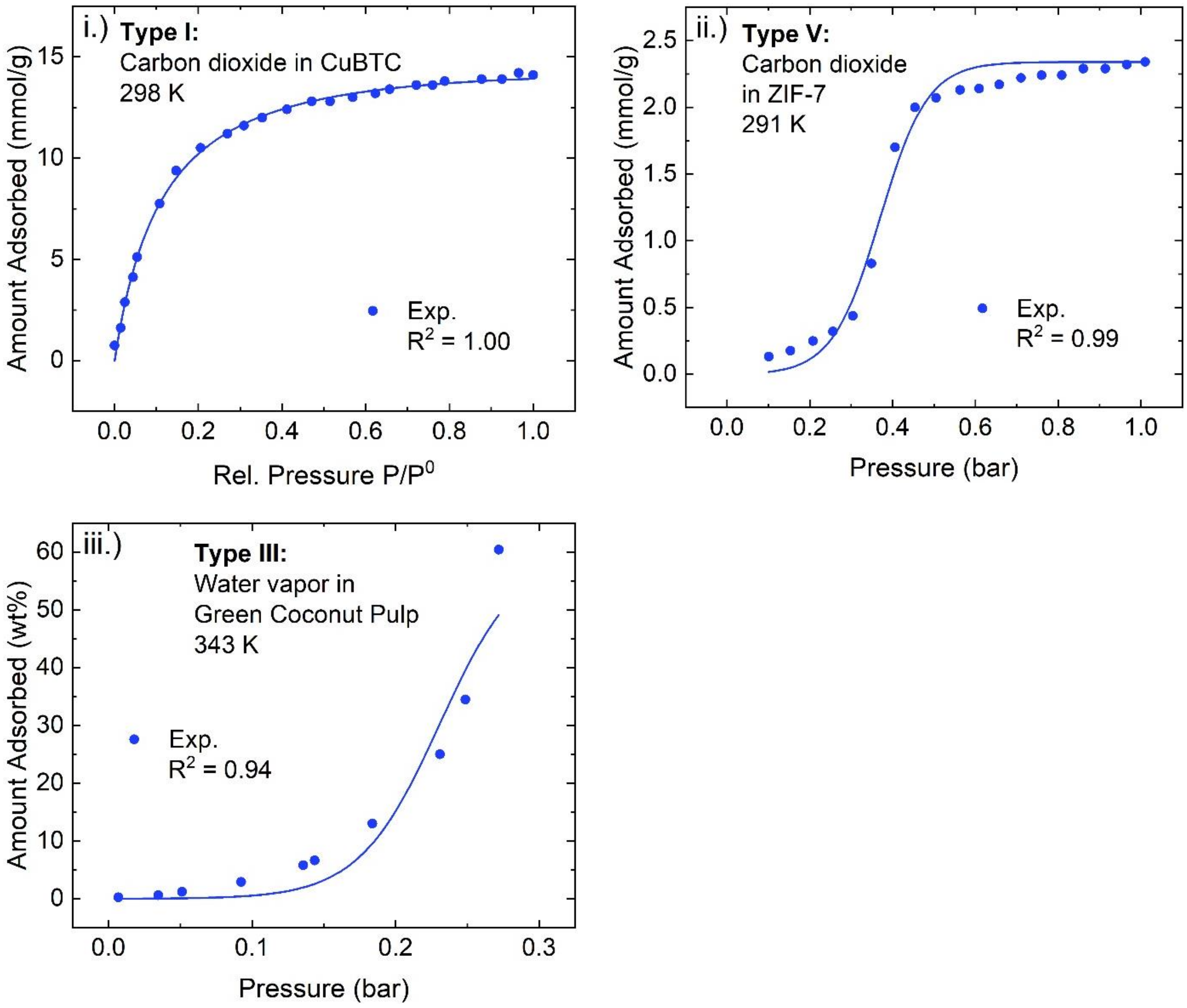

Figure S2. Fits of experimental data from the literature in absolute units using the model presented in this manuscript from Figure 1B for (i.) carbon dioxide in CuBTC at 298 K[1], (ii.) carbon dioxide in ZIF-7 at 291 K[2], and (iii.) water vapor in Green Coconut Pulp at 343 K[3]. The fitting parameter are listed in Table S4.

Table S1. Fitting parameters, references, and coefficients of determination for literature examples presented in Figure S1.

# Ref.	Adsorbate	Adsorbent	Parameter a [Pressure^{-1}]	Parameter b [Pressure^{-1}]	Parameter q_{max} [Loading]	R^2
i.)[4]	N_2	UiO-66	9.650E+02	-1245	389	0.2503
ii.)[5]	N_2	CS-VTM-2.0	3.044E-01	2.667	0.529	0.8271
iii.)[6]	H_2O	SBA-15	2.536E-07	22.48	0.850	0.9590
iv.)[7]	CO_2	MIL-53(Fe)	1.956E-02	0.027	3.65	0.8854
v.)[6]	H_2O	FSM-16	1.715E-07	28.48	0.922	0.9721
vi.)[6]	H_2O	MCM-41	1.677E-07	27.40	0.717	0.9429
vii.)[8]	H_2O	MIL-101(Cr)	4.192E-04	23.02	75.85	0.9942
iix.)[9]	CH_4	MgO	2.867E-02	0.339	352	0.8325
ix.)[10]	Kr	Carbopack F	4.683E-01	0.910	48.7	0.8331

Table S2. Summary of isotherm models published in literature describing type V isotherms[11]. The application of these existing models to describe type V isotherms are shown in additional citations to the specific models. The symbols are adopted from the original publications.

Name[ref.]	Model	# Fitting Parameter	Application System
Dubinin-Astakhov (1971)[12-13]	$N_a = N_{a0} \cdot exp\left[-\left(\frac{RT \cdot ln\left(p_0/_0\right)}{E}\right)^n\right]$	2	Water vapor in active carbon
Dubinin-Serpinsky (1981)[14]	$h = a \Big/ \left[c(a_0 + a)\left(1 - \frac{c-1}{c} \cdot \frac{a}{a_1}\right)\right]$	3	Water vapor in microporous carbonaceous adsorbents
Ruthven (1984)[15]	$q = \frac{q_s(K^a + 2K^b p)p}{(1 + K^a p + K^b p^2)}$	2	General S-shaped isotherms (second-order equation of general statistical thermodynamics isotherm)
Mahle-Friday (1989)[16]	$q = \frac{V_m}{V_L}\left[1 - \frac{\Gamma(p+1,\epsilon)}{\Gamma(p+1)}\right] + \frac{V_m}{V_L}\left[1 - \frac{\Gamma(p'+1,\epsilon')}{\Gamma(p'+1)}\right]$<br>$\epsilon = \alpha r$ and $\epsilon' = \alpha' r$	5	Water vapor on microporous carbons
Barton-Evans-MacDonald (1991)[17]	$h = \frac{a}{ca_0 + ca(1 - \exp(-k^2(a - a_c)^2))}$	3	Water vapor in porous carbon
Talu-Meunier (1996)[18]	$P = \frac{H\Psi}{1 + K\Psi} \cdot \exp\left(\frac{\Psi}{N_m}\right)$<br>$\Psi = \frac{-1 + \sqrt{1 + 4K \cdot \frac{N_m \cdot N}{N_m - N}}}{2K}$	2	Water vapor on carbon

Continued **Table S2.** Summary of isotherm models published in literature describing type V isotherms[11]. The application of these existing models to describe type V isotherms are shown in additional citations to the specific models. The symbols are adopted from the original publications.

**Name**[ref.]	**Model**	**# Fitting Parameter**	**Application System**
Do-Do (2000)[19]	$C_\mu = C_{\mu,s}\frac{K_\mu \sum_{n-6} x^n}{K_\mu \sum_{n-6} x^n + \sum_{n-6} x^{n-5}} + S_0 \frac{K_f \sum_{n-1} n x^n}{1+K_f \sum_{n-1} x^n}$	3	Water vapor in activated carbon
Mahle (2002)[20]	$\frac{n}{n_s} = \frac{1}{D}\left(\tan^{-1}\left(\frac{p^*-A}{B}\right) - \tan^{-1}\left(\frac{-A}{B}\right)\right)$ $D = \tan^{-1}\left(\frac{1-A}{B}\right) - \tan^{-1}\left(\frac{-A}{B}\right)$	2	Water vapor on activated carbon
Chakraborty-Sun (2014)[21]	$\theta = \frac{\beta\left(\frac{p}{\varphi^*}\right)}{\left[1+C\left(\frac{p}{\varphi^*}\right)^m\right]^{1/m}}$ $C = \beta^m - \left[\frac{1}{exp(z)}\right]^m$ and $\varphi^* = P_s \cdot exp(-z)$	3	General multi-type adsorption
Ng-Burhan-Shahzad-Ismail (2017)[22]	$\theta_t = \alpha_1\left[\frac{\left(\frac{p}{p_s}exp\left(\frac{\epsilon_{01}}{RT}\right)\right)^{\frac{RT}{m_1}}}{1+\left(\frac{p}{p_s}exp\left(\frac{\epsilon_{01}}{RT}\right)\right)^{\frac{RT}{m_1}}}\right] + \alpha_2\left[\frac{\left(\frac{p}{p_s}exp\left(\frac{\epsilon_{02}}{RT}\right)\right)^{\frac{RT}{m_2}}}{1+\left(\frac{p}{p_s}exp\left(\frac{\epsilon_{02}}{RT}\right)\right)^{\frac{RT}{m_2}}}\right]$	4	General adsorption on porous heterogeneous surfaces
Butyrskaya-Zapryagaev (2021)[23]	$q = q_m \frac{b_1 P + \frac{2}{m_2} b_2 P^2 + \ldots \frac{i}{m_i} b_i P^i \ldots + \frac{n}{m_n} b_n P^n}{1+b_1 P + b_2 P^2 + \ldots b_i P^i \ldots + b_n P^n}$	>3	CO_2 adsorption in IRMOF-1

Table S3. Fitting parameter for the fits of the six general IUPAC isotherm types shown in Figure 1A.

Type	Parameter a [-]	Parameter b [-]	Parameter q_{max} [-]	R^2
I	23.38	-20.28	0.614	0.9997
II	1.767	-0.063	0.906	0.9219
III	2.048E-4	14.25	0.869	0.9943
IV	0.500	3.966	0.913	0.8738
V	6.741E-4	15.13	0.826	0.9998
VI	1.090	1.918	0.421	0.9167

Table S4. Fitting parameter for the fits of the three experimental isotherms of isotherm types I, III, and V shown normalized in Figure 1B and not normalized in Figure S2.

Type	Adsorbate	Adsorbent	Parameter a [$Pressure^{-1}$]	Parameter b [$Pressure^{-1}$]	Parameter q_{max} [Loading]	R^2
I	CO_2	CuBTC	9.625E+00	-6.922	14.20	0.9976
V	CO_2	ZIF-7	2.756E-02	17.42	2.340	0.9897
III	H_2O	Green Coconut Pulp	9.547E-03	35.65	60.42	0.9408

Table S5. Fitting parameter for the fits of the water isotherms in the five materials with different hydrophilicities shown in Figure 2B and plotted in the bar chart in Figure 2C.

Adsorbent	Parameter a [-]	Parameter b [-]	Parameter q_{max} [g/g]	R^2
Mg-MOF-74	5.815E+01	-58.76	0.633	0.9933
13X	1.679E+02	-172.68	0.217	0.9821
KIT-1	6.436E-07	28.79	0.839	0.9855
MCM-48	1.092E-07	27.97	0.844	0.9433
SBA-1	4.079E-05	28.57	0.444	0.9836

Table S6. Fitting parameter for the fits of the water isotherms in the five MCM-41 derivatives shown in Figure 3A.

MCM	Parameter a [-]	Parameter b [-]	Parameter q_{max} [g/g]	R^2
10	1.928E-02	19.42	0.305	0.9966
12	1.924E-05	30.40	0.460	0.9862
14	1.924E-07	33.40	0.561	0.9853
16	1.028E-08	34.42	0.700	0.9954
18	7.228E-10	36.22	0.850	0.9903

Table S7. Fitting parameter for the fits of the water isotherms in four carbon materials where specific models have been developed in the literature shown in Figure 3B. The coefficient of determination for the two fits are compared between the models reported in the literature and the models presented in this work.

**Carbon**	**Parameter a [-]**	**Parameter b [-]**	**Parameter q_{max} [mol/g]**	**$R^2_{This\ work}$**	**R^2_{Lit}**
AX-21	1.805E-09	32.70	52.50	0.993	0.991[18]
N-125	1.580E-05	21.72	25.88	0.996	0.995[13]
AC7	5.973E-02	8.825	27.10	0.997	1.000[14]
BPL	1.672E-02	12.06	21.50	0.993	0.997[20]

Table S8. Fitting parameter for the fits of the methane isotherms in CdIF-13 at three different temperatures as shown in Figure 4A and also tabulated in the inset in Figure 4B.

**T [°C]**	**Parameter a [bar^{-1}]**	**Parameter b [bar^{-1}]**	**Parameter q_{max} [mmol/g]**	**R^2**
-20	2.058E-14	1.170	4.99	0.9970
-10	1.658E-14	0.865	5.00	0.9987
0	9.430E-13	0.581	4.89	0.9899

Table S9. Fitting parameter for the fits of the carbon dioxide and oxygen isotherms in $Cu(dhbc)_2(4,4'bpy)$ at 298 K as shown in Figure 5A.

**#**	**Parameter a [MPa^{-1}]**	**Parameter b [MPa^{-1}]**	**Parameter q_{max} [mol/mol]**	**R^2**
CO_2	9.695E+00	-10.05	2.77	0.9835
O_2	8.094E-5	2.214	1.85	0.9995

REFERENCES

1. Al-Janabi, N.; Hill, P.; Torrente-Murciano, L.; Garforth, A.; Gorgojo, P.; Siperstein, F.; Fan, X., Mapping the Cu-BTC metal–organic framework (HKUST-1) stability envelope in the presence of water vapour for CO_2 adsorption from flue gases. *Chem. Eng. J.* **2015,** *281*, 669-677.

2. Kamali, K.; Prasad, S.; Sahoo, M. K.; Behera, J. N.; Waghmare, U. V.; Narayana, C., Unusual CO_2 Adsorption in ZIF-7: Insight from Raman Spectroscopy and Computational Studies. *Inorg. Chem.* **2022,** *61*, 11571-11580.

3. Burhan, M.; Shahzad, M. W.; Ng, K. C., Energy distribution function based universal adsorption isotherm model for all types of isotherm. *Int. J. Low Carbon Technol.* **2018,** *13*, 292-297.

4. Cmarik, G. E.; Kim, M.; Cohen, S. M.; Walton, K. S., Tuning the Adsorption Properties of UiO-66 via Ligand Functionalization. *Langmuir* **2012,** *28*, 15606-15613.

5. Zhang, W.; Lan, Y.; Ma, M.; Chai, S.; Zuo, Q.; Kim, K.-H.; Gao, Y., A novel chitosan–vanadium-titanium-magnetite composite as a superior adsorbent for organic dyes in wastewater. *Environ. Int.* **2020,** *142*, 105798.

6. Ng, E.-P.; Mintova, S., Nanoporous materials with enhanced hydrophilicity and high water sorption capacity. *Microporous Mesoporous Mater.* **2008,** *114*, 1-26.

7. Schneemann, A.; Bon, V.; Schwedler, I.; Senkovska, I.; Kaskel, S.; Fischer, R. A., Flexible metal–organic frameworks. *Chem. Soc. Rev.* **2014,** *43*, 6062-6096.

8. Küsgens, P.; Rose, M.; Senkovska, I.; Fröde, H.; Henschel, A.; Siegle, S.; Kaskel, S., Characterization of metal-organic frameworks by water adsorption. *Microporous Mesoporous Mater.* **2009,** *120*, 325-330.

9. Gay, J. M.; Suzanne, J.; Coulomb, J. P., Wetting, surface melting, and freezing of thin films of methane adsorbed on MgO(100). *Phys. Rev. B* **1990,** *41*, 11346-11351.

10. Prasetyo, L.; Horikawa, T.; Phadungbut, P.; Tan, S.; Do, D. D.; Nicholson, D., A GCMC simulation and experimental study of krypton adsorption/desorption hysteresis on a graphite surface. *J. Colloid Interface Sci.* **2016,** *478*, 402-412.

11. Mozaffari Majd, M.; Kordzadeh-Kermani, V.; Ghalandari, V.; Askari, A.; Sillanpää, M., Adsorption isotherm models: A comprehensive and systematic review (2010−2020). *Sci. Total Environ.* **2022,** *812*, 151334.

12. Dubinin, M. M.; Astakhov, V. A., Development of the concepts of volume filling of micropores in the adsorption of gases and vapors by microporous adsorbents. *Bull. Acad. Sci. USSR, Div. Chem. Sci.* **1971,** *20*, 3-7.

13. Stoeckli, F.; Jakubov, T.; Lavanchy, A., Water adsorption in active carbons described by the Dubinin–Astakhov equation. *J. Chem. Soc., Faraday Trans.* **1994,** *90*, 783-786.

14. Dubinin, M. M.; Serpinsky, V. V., Isotherm equation for water vapor adsorption by microporous carbonaceous adsorbents. *Carbon* **1981,** *19*, 136.

15. Ruthven, D. M., *Principles of adsorption and adsorption processes*. John Wiley and Sons, Inc.: New York (USA), 1984.

16. Mahle, J. J.; Friday, D. K., Water adsorption equilibria on microporous carbons correlated using a modification to the Sircar isotherm. *Carbon* **1989,** *27*, 835-843.

17. Barton, S.; Evans, M.; MacDonald, J., An equation describing water vapour absorption on porous carbon. *Carbon* **1992,** *30*, 123-124.

18. Talu, O.; Meunier, F., Adsorption of associating molecules in micropores and application to water on carbon. *AIChE J.* **1996,** *42*, 809-819.

19. Do, D.; Do, H., A model for water adsorption in activated carbon. *Carbon* **2000,** *38*, 767-773.

20. Mahle, J. J., An adsorption equilibrium model for Type 5 isotherms. *Carbon* **2002,** *40*, 2753-2759.

21. Chakraborty, A.; Sun, B., An adsorption isotherm equation for multi-types adsorption with thermodynamic correctness. *Appl. Therm. Eng.* **2014,** *72*, 190-199.

22. Ng, K. C.; Burhan, M.; Shahzad, M. W.; Ismail, A. B., A Universal Isotherm Model to Capture Adsorption Uptake and Energy Distribution of Porous Heterogeneous Surface. *Sci. Rep.* **2017,** *7*, 10634.

23. Butyrskaya, E. V.; Zapryagaev, S. A., Cluster model of the step-shaped adsorption isotherm in metal–organic frameworks. *Microporous Mesoporous Mater.* **2021,** *322*, 111146.
